# Generation of two-cell cloned embryos from mouse faecal cell

**DOI:** 10.1038/s41598-018-33304-2

**Published:** 2018-10-08

**Authors:** Satoshi Kamimura, Sayaka Wakayama, Hiroki Kuwayama, Yoshiaki Tanabe, Satoshi Kishigami, Teruhiko Wakayama

**Affiliations:** 1 Faculty of Life and Environmental Sciences, University of Yamanashi, Yamanashi, 400–8510 Japan; 2 Advanced Biotechnology Centre, University of Yamanashi, Yamanashi, 400–8510 Japan; 3 Present Address: Department of Basic Medical Sciences for Radiation Damages, National Institute of Radiological Sciences, Chiba, 263–8555 Japan

## Abstract

Cloning animals using nuclear transfer (NT) provides the opportunity to preserve endangered species. However, there are risks associated with the collection of donor cells from a body, which may cause accidental death of the animal. Here, we tried to collect faeces-derived cells and examined the usability of those nuclei as a donor for NT. A relatively large number of cells could be collected from GFP-Tg mouse faeces by this method. After NT, only 4.2% of the reconstructed oocytes formed pseudo-pronucleus. This rate increased up to 25% when GFP and Hoechst were used as a marker to select better cells. However, the reconstructed oocytes/embryos showed several abnormalities, such as shrunken nuclear membranes and abnormal distribution of tubulin, and none of them developed beyond one-cell stage embryos. These developmental failures were caused by not only toxic substances derived from faeces but also intrinsic DNA damage of donor cell nuclei. However, when the serial NT was performed, some of the cloned embryos could develop to the two-cell stage. This method may remove toxic substances and enhance DNA repair in the oocyte cytoplasm. Thus, these results indicate that faeces cells might be useful for the conservation of endangered species when technical improvements are achieved.

## Introduction

The nuclear transfer (NT) technique is expected to be applied in various fields such as regenerative medicine, preparation of biological products, livestock production, and species conservation^1,2^. Especially in species conservation, the NT technique has the potential for a resurgence of extinct species and rescue of endangered species. However, in endangered species, individuals are rare and valuable, and it is difficult to obtain donor cells for NT from these animals. In addition, many of these animals are usually protected from hunting by protection treaties and laws in each country.

To avoid the risk of injury or accidental death at the donor cell collection from rare/endangered animals, several methods have been reported. For example, cloned mice could be generated using leucocytes derived from a drop of peripheral blood with low-invasiveness^3^. However, this method is unsuitable for wild endangered animals because it is not easy handled without anesthesia treatment. On the other hand, cloned mice and cows were generated from urine-derived cells, which was a completely noninvasive method^4,5^. This method is useful for zoo animals because it is relatively easy to collect fresh and clean urine. However, it is still difficult to apply to wild, endangered animals because the urine seeps down into the ground immediately before collection.

By contrast, faeces can be collect noninvasively and more easily even from wild animals. Previous reports demonstrated that DNA could be extracted from faeces^6–8^ and that DNA was used to clarify genetic diversity, evolutionary processes of natural populations and behavioral ecology including kinship analysis in some wild spices^9–11^. If the nucleus derived from faeces can be shown to be suitable as nuclear donors, then it will be very useful in generating cloned animals from wild endangered species without harming them.

In this study, we examined whether the donor cells can be collected from faeces to generate cloned animals.

## Results

### Collection of cell-like bodies (CLBs) from mouse faeces

In the preliminary experiment, the whole faeces of 129-GFP mice were dissolved and suspended in phosphate buffered saline (PBS). The faecal suspensions were washed with PBS by centrifugation and then observed under a microscope. However, no cells could not be picked up from the suspension because numerous debris existed. To separate and remove the debris, different concentrations of Ficoll solution were used; however, we could not find suitable conditions for this procedure. We gave up on isolating faecal cells using this methods.

**Figure 1:**
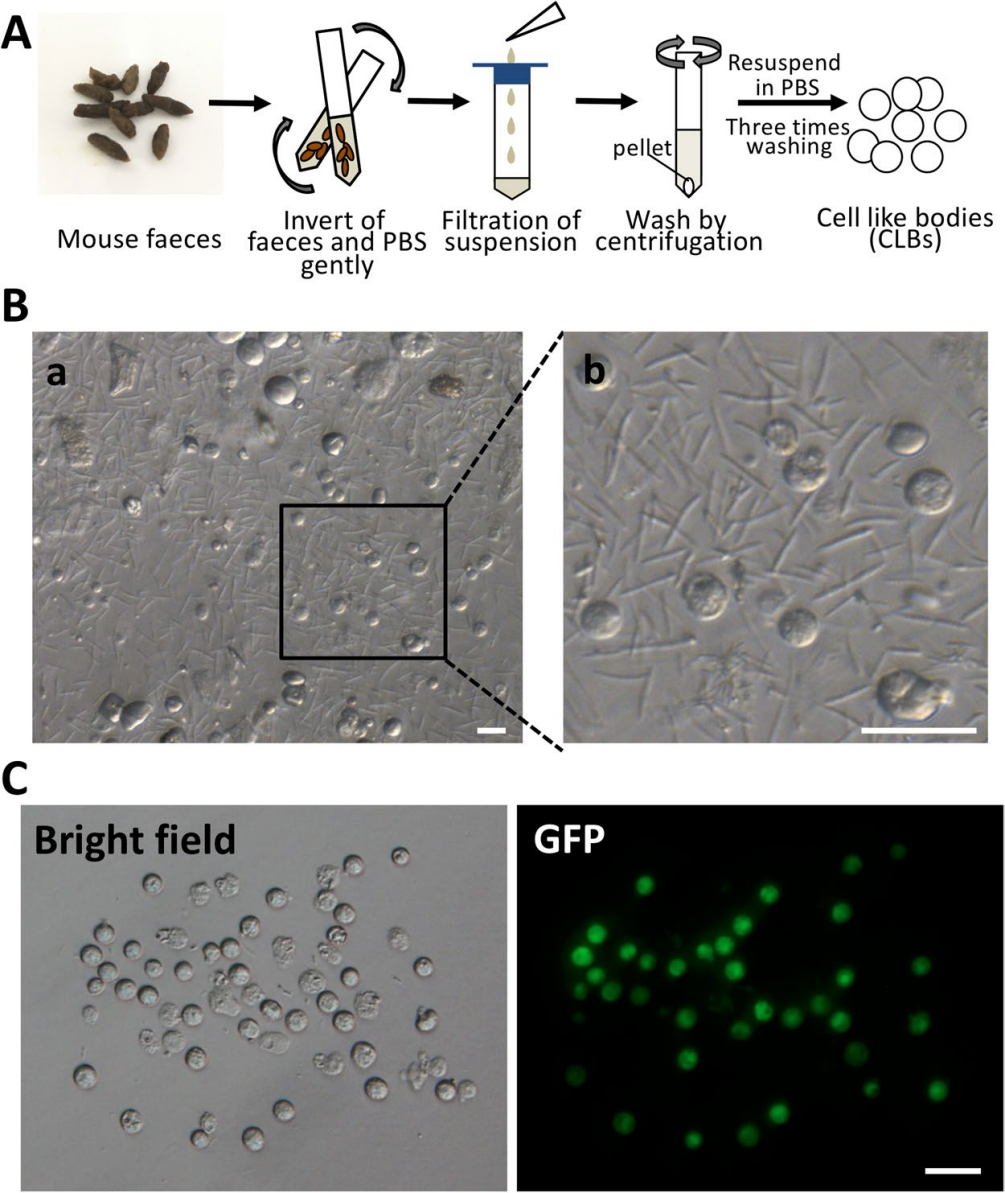
Collection of cell-like bodies from mouse faeces. (**A**) Scheme of a collection of cell-like bodies. (**B**) Faecal suspension derived from 129-GFP mouse (a) and its extended image (b). Many cell-like bodies (CLBs) existed in faecal suspension. (**C**) The CLBs were collected from a faecal suspension using a micromanipulator and have GFP. Bar = 20 μm.

Then, we tried to collect faecal cells from the surface of faeces. If cells existed on the faeces, then those cells must have been epidermal cells of the intestine, and those cells were probably attached to the surface of faeces. The collected faeces were immersed in PBS solution in 15 ml centrifuge tube for five minutes and gently tapped and inverted several times (Fig. 1A). Then, the supernatants were filtered and moved to another tube and centrifuged for 5 min at 280 × g. The pellets were resuspended with PBS and repeated two more times. Although there were still substantial debris and small bacteria, a “cell-like round sphere”, which has a similar size and shape as normal cells, could be observed in the suspension (Fig. 1Ba,Bb). Those spheres could be easily collected from debris using micropipettes attached with a micromanipulator (Fig. 1Bc). However, when we observed the GFP expression of those spheres, some of them did not express GFP even when all faeces were collected from GFP-Tg mouse strain. In those cells, the cell membrane was likely broken, and GFP leaked out of the cytoplasm. However, we cannot discard the possibility that the some of them were not cells. Therefore, we described those that may not be cells as cell-like bodies (CLBs).

### Examination of CLBs

**Figure 2:**
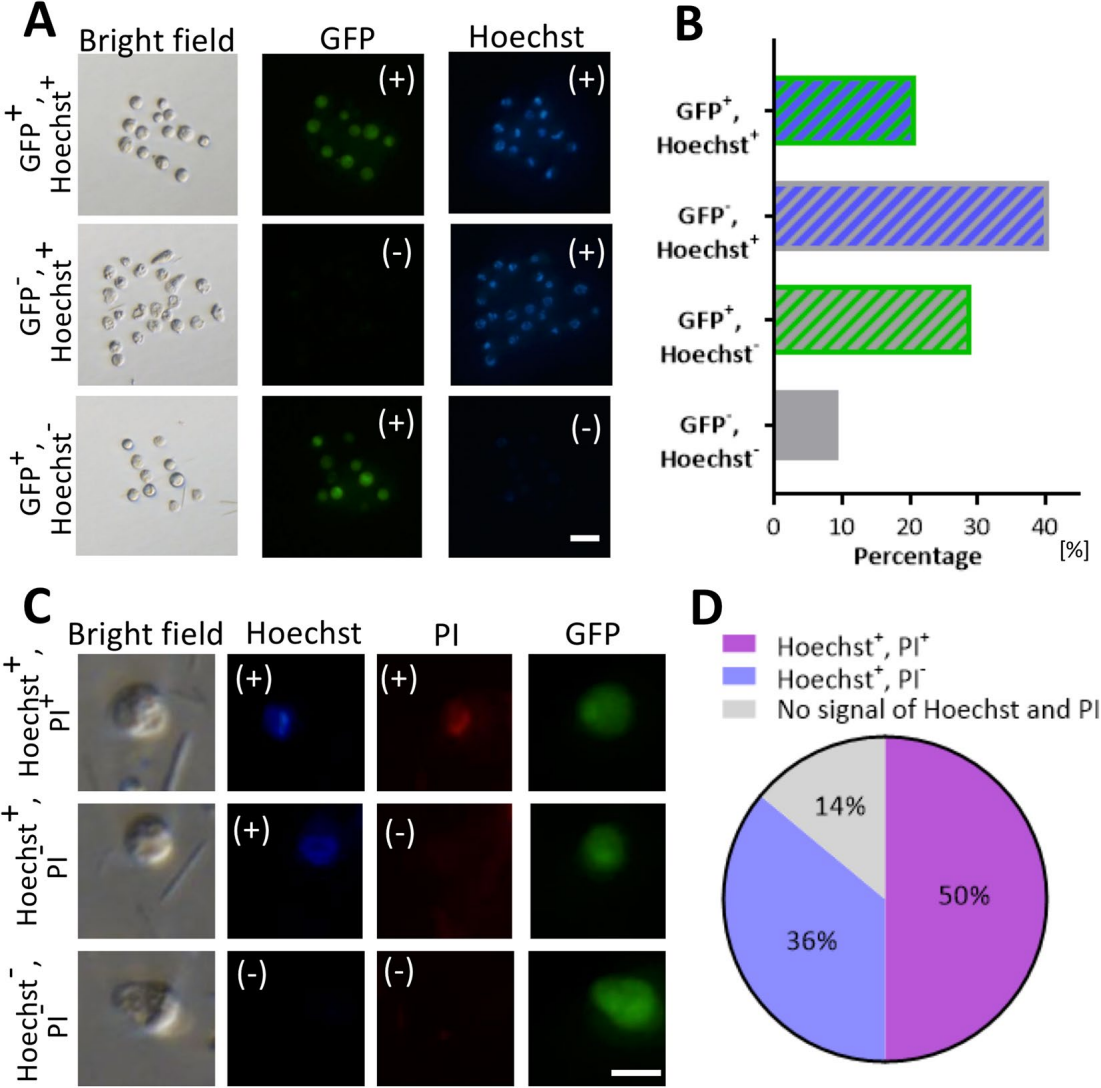
Observation of cell-like bodies for nuclear transfer from the faecal suspension. (**A**) Hoechst staining of CLBs and different staining pattern of Hoechst and GFP signal. Bar = 20 μm. (**B**) Percentage of CLB population with each Hoechst and GFP signal patterns were quantified in images (A). (**C**) Costaining of Hoechst and PI for CLBs and observations with these and GFP signal. Bar = 50 μm. (**D**) Percentage of CLB population with different Hoechst, PI and GFP signal patterns were quantified in images (**C**).

From the above study, we hypothesized that some of CLBs were not real cells. We characterized the CLBs by staining with Hoechst to detect the nucleus. As a result, more than half of CLBs possessed a nucleus irrespective of GFP expression (Fig. 2A,B, GFP-positive: 21%; GFP-negative 41%). On the other hand, 30% of the CLBs expressed GFP but did not have a nucleus inside the body (Fig. 2A,B).

Next, we tried to detect whether those CLBs had intact cell membranes. CLBs were costained with Hoechst and PI. Hoechst can stain all cell nuclei irrespective of cell condition, whereas PI can stain cell nuclei only when cell membrane is broken. As a result, 50% of CLBs were positive, which suggest that those cell membranes were broken (Fig. 2C,D). Approximately 36% of CLBs were Hoechst-positive and PI-negative, which suggests that those cells have an intact cell membrane. The remaining CLBs had no nucleus.

As shown in Fig. 2A, GFP^−^/Hoechst^+^ CLBs had distorted morphologies on the cell surface and no clear outline of the cell membrane under the bright field conditions compared to those with GFP ^+^/Hoechst^+^. On the other hand, GFP ^+^/Hoechst^+^ and GFP ^+^/Hoechst^-^ CLBs were spherical forms, the outline of the cell membrane was clear and both sizes were the same. This observation suggests that it is difficult to distinguish between GFP^+^/Hoechst^+^ and GFP^+^/Hoechst^−^ under the bright field conditions.

### Potential of pronuclear formation on faecal cell cloned embryos

**Figure 3:**
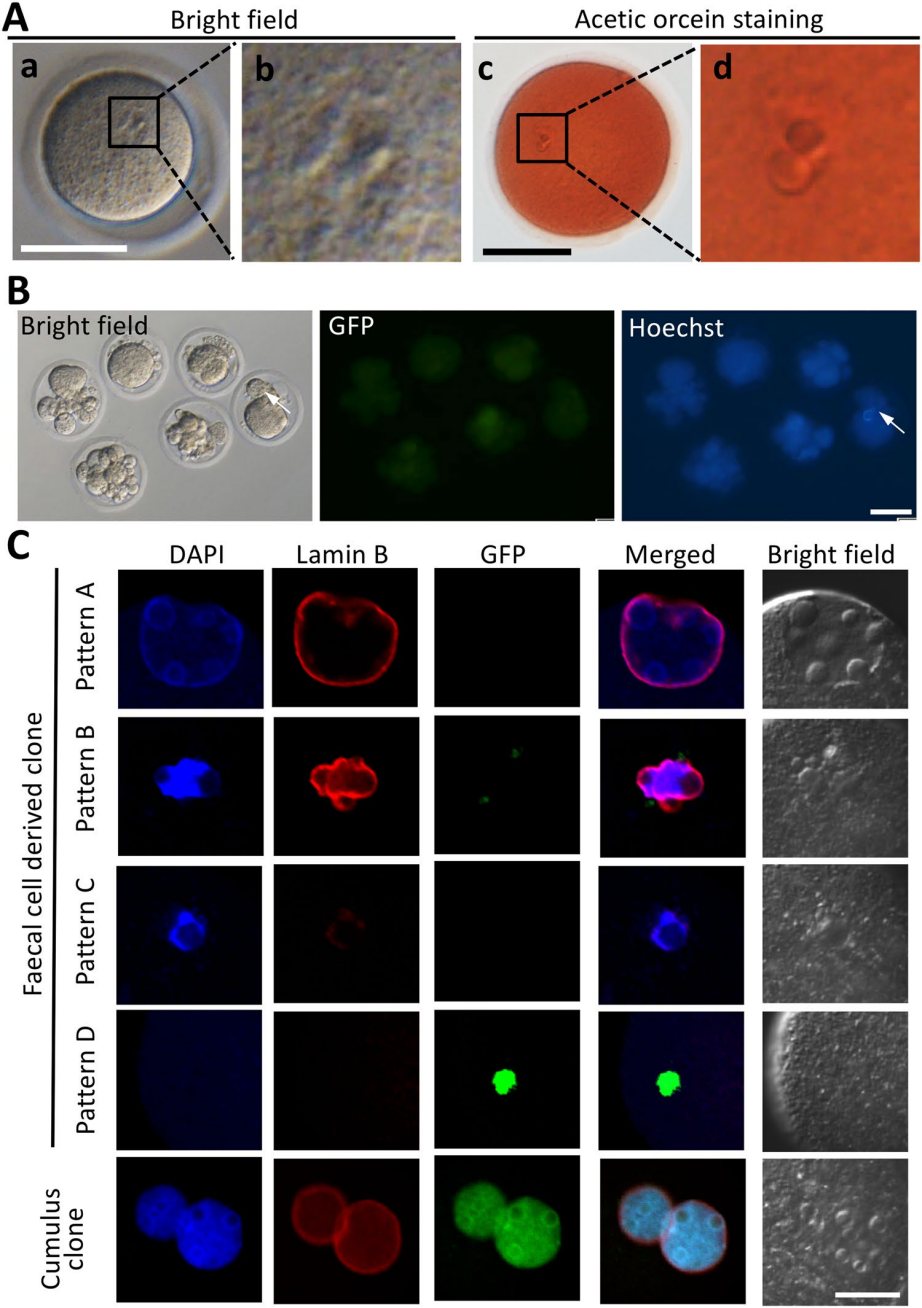
Pronuclear formation and developmental potential of cloned embryos derived from the faecal cell. (**A**) Pronuclear formation of faeces-derived clone. The randomly chosen CLBs were injected into enucleated oocytes, and then, these embryos were observed under the microscope at 8 hpa (a and b). Then, the embryos were stained with acetic orcein (c and d), and 4.2% of the embryos showed the pronuclear formation (see also Table 1). Bar = 50 μm. (**B**) Culture of faeces-derived clone up to 4 days. All embryos were arrested at one-cell or fragmentation despite some embryos being Hoechst-positive (arrow). Bar = 50 μm. (**C**) The cloned embryos at 8 hpa were immunostained to detect nuclei and nuclear membranes using DAPI and anti-lamin B antibodies, respectively. Four patterns of pronuclear formation (pattern A–D) were shown in faeces-derived clones (see text for detailed description of each pattern). Embryo numbers of each pattern were quantified in Table 2. The pronuclei of cumulus clones were also observed as a control. Bar = 20 μm.

When randomly selected CLBs were injected into enucleated oocytes and the reconstructed oocyte were activated, only a few cloned embryos formed a pseudo-pronucleus (4.2%, 2/48) (Fig. 3A). Then, CLBs were divided into three group according to the GFP and Hoechst expression pattern (Fig. 2A) and injected into enucleated oocytes separately. As a result, pronuclear formation increased up to 25% when GFP and Hoechst double positive CLBs were used (Table 1). The size of the pronucleus relatively increased compared to that with a randomly selected injection. When GFP-negative and Hoechst-positive CLBs were used, the pronuclear formation rate decreased (13.0%). GFP-positive but Hoechst-negative CLBs did not show any pronuclear formation. For the following experiments, we used only Hoechst-positive CLBs for nuclear transfer.

**Table 1:** Pronuclear formation and developmental potential of cloned embryos using faeces-derived cells.

Donor cell type	Donor cell group	No. of constructed embryos after donor cell injection	No. of PN formation at 8 hpa (%)	No. of embryos at 24 hpa (%)			
				2-cell	1-cell	Fragmented	Died
Faeces-derived cell	GFP^+^, Hoechst^+^	20	5 (25.0)	0 (0)	1 (5.0)	19 (95.0)	0 (0)
	GFP^−^, Hoechst^+^	23	3 (13.0)	0 (0)	5 (21.7)	17 (73.9)	1 (4.3)
	GFP^+^, Hoechst^−^	25	0 (0)	0 (0)	9 (36.0)	16 (64.0)	0 (0)

However, when reconstructed embryos derived from CLBs were cultured up to the next day, most of the embryos were fragmented, and none of the embryos developed to the 2-cell stage, irrespective of GFP expression (Table 1 and Fig. 3B).

### Morphology of pronucleus in faeces cloned embryos

Next, the pronuclear morphology of cloned embryos derived from CLBs was observed by staining of the nuclear membrane using anti-lamin B antibody. As a result, it was found that faeces-derived clone was divided into the following patterns (Fig. 3C): Pattern A: the nuclear membrane was shown as a clear and circular shape, and the nucleus and nucleolus can be shown as the same as the cumulus clone; Pattern B: the nucleus and the nuclear membrane were clear, but the collapsed pronuclear-like structure could be shown. Pattern C: the nuclear membrane signal was ambiguous or could not detect, but the nucleus was observed; and Pattern D: both nucleus and nuclear membrane signals could not be shown. As shown in Table 2, a relatively normal embryo (pattern A) was obtained when GFP^−^/Hoechst^+^ CLBs were injected into oocytes, but the efficiency was low (10.5%). Approximately 20% of embryos belong to pattern B, and 70% of embryos belong to pattern C, irrespective of the GFP expression of Hoechst-positive donor cells.

**Table 2:** Pronuclear formation pattern of cloned embryos derived from faecal cell nucleus.

Donor cell type	Donor cell group	No. of cloned embryos performed immunostaining	No. of each pattern of pronuclear formation at 8 hpa (%)			
			Pattern A	Pattern B	Pattern C	Pattern D
Faeces-derived cell	GFP^+^, Hoechst^+^	18	1 (5.6)	4 (22.2)	13 (72.3)	0 (0)
	GFP^−^, Hoechst^+^	19	2 (10.5)	4 (21.1)	13 (68.4)	0 (0)
	GFP^+^, Hoechst^−^	13	0 (0)	0 (0)	1 (7.7)	12 (92.3)
Cumulus cell	—	11	11 (100)	0 (0)	0 (0)	0 (0)

### Premature chromosome condensation (PCC) and spindle formation of faecal cell-derived donor nucleus in reconstructed oocytes

**Figure 4:**
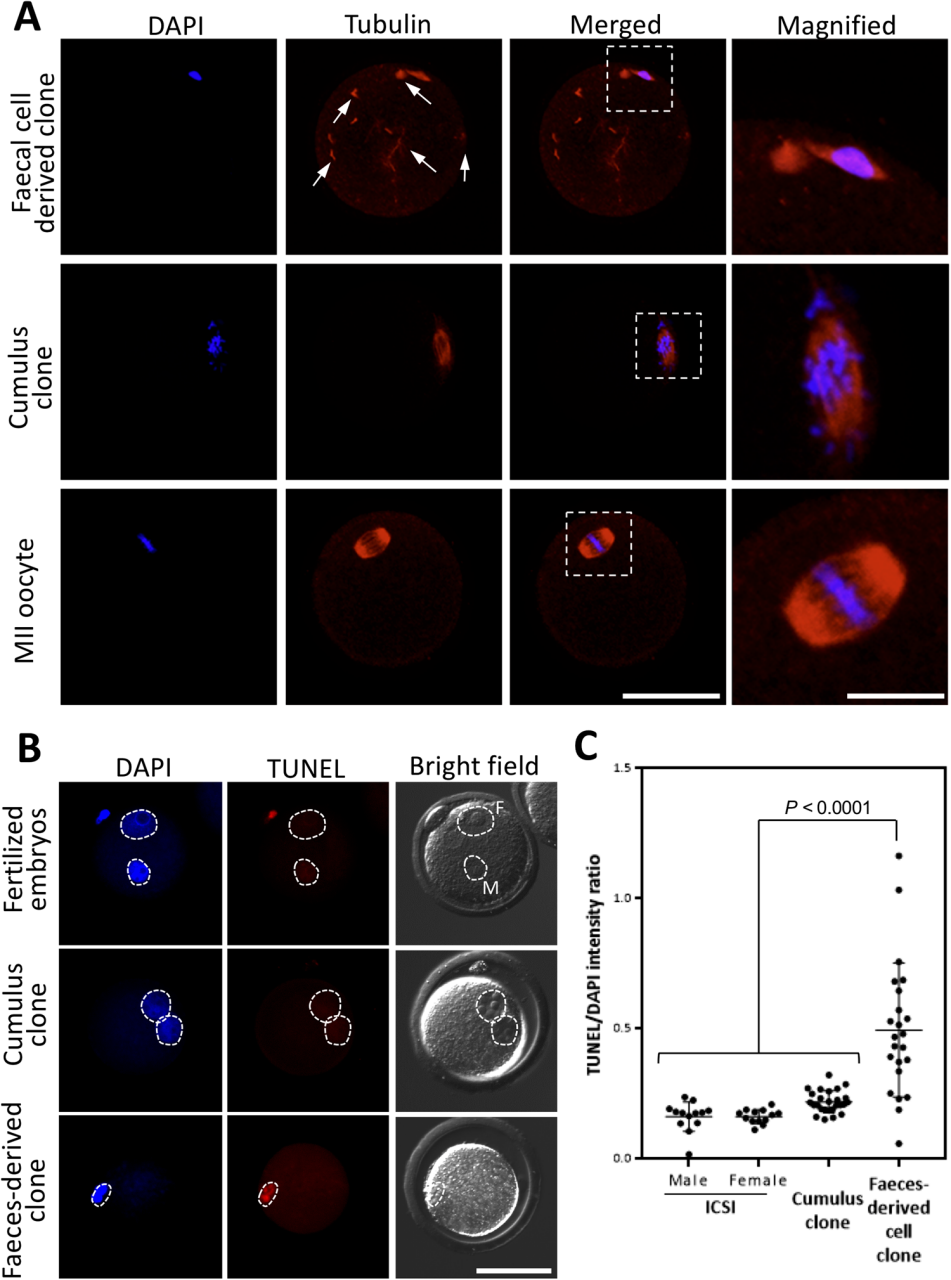
Observation of tubulin of reconstructed oocytes and DNA damage of pronucleus of embryo derived from faecal cell. (**A**) The reconstructed embryos injected with faecal cells after 2 hr were immunostained to observe the tubulin distribution, and the DNA was stained using DAPI. As a control, embryos were reconstructed using cumulus cell and MII oocytes. In the faecal cell derived clone, ectopic tubulin localization was detected (arrow head), and PCC was hard to observe compared with cumulus clones and MII oocytes. Bar = 50 μm. The rightmost panels show magnified views of the region indicated by the white dashed box. Bar = 15 μm. (**B**) Each embryo collected at 8 hpa or 8 hpi had a TUNEL assay conducted with DAPI staining for nuclear detection. Dotted circles indicate the pronucleus. F and M in fertilized embryos show female and male pronuclei, respectively. Bar = 50 μm. (**C**) Relative fluorescent intensity of TUNEL per DAPI. One dot indicates the intensity of one pronuclei. The relative intensity of faeces-derived clones was significantly higher than fertilized embryos and cumulus cloned embryos (Tukey’s test, *P* < 0.0001). n = numbers of embryos tested.

We hypothesized that the low pronuclear formation rate in CLBs derived cloned embryos is the failure of PCC and spindle formation of donor nucleus in reconstructed oocytes. To exam it, reconstructed oocytes were observed without activation, and PCC and tubulin were observed by DAPI and immunostaining, respectively. When reconstructed oocytes were observed, only 4.5% (1/22) of oocytes showed relatively normal PCC and spindle formation. Most of the oocytes failed PCC and spindle formation (95.5%, 21/22). Those failed oocytes showed ectopically or scattered tubulin distribution inside oocytes cytoplasm (63.6%, 14/22) (Fig. 4A, arrow).

### Toxicity of faecal substances on embryo development

These abnormalities might be caused by faeces derived substances, which may bind with donor nuclei and were injected it into oocytes at the same time. To test this hypothesis, the supernatant of faecal suspension were injected into intact oocytes, and the developmental potential was observed after parthenogenetic activation. In untreated and PBS- injected parthenogenetic embryos, the rate of blastocyst formation was 94.3% and 76.7%, respectively (Table 3), whereas when a small amount of supernatant (approximately 10 pl) was injected into MII oocyte and activated, although most of them showed normal pronuclear formation, the development of the 2-cell stage embryo remarkably decreased, and the rate of blastocyst formation was only 2%. In addition, when faecal cell nuclei were injected into intact oocytes and activated, most of them formed female pronucleus, but none of them developed to the blastocyst stage. These results suggest that toxic substances derived from faeces caused developmental failure of cloned embryos.

**Table 3:** Development of parthenogenetic embryos injected with faecal cell nucleus or suspension.

	Injection material	No. of oocytes	No. of PN formation at 8 hpa (%)	No. of embryos at 24 hpa (%)				No. of embryos at 48 hpa (%)	No. of embryos at 72 hpa (%)	No. of embryos at 96 hpa (%)
				2-cell	1-cell	Fragmented	Died	4–8 cell	Morulae/ Blastocyst	Blastocyst
Parthenogenesis after injection into MII oocyte	faecal cell (Hoechst+)	70	68 (97.1)	7 (10.3)	39 (57.4)	21 (30.9)	3 (4.4)	2 (2.9)	0 (0)	0 (0)
	faecal suspension	44	43 (97.7)	8 (18.6)	33 (76.7)	3 (7.0)	0 (0)	3 (7.0)	3 (7.0)	1 (2.3)
	PBS	45	43 (95.6)	40 (93.0)	3 (7.0)	2 (4.7)	0 (0)	39 (90.7)	38 (88.4)	33 (76.7)
Parthenogenesis (control)	none	40	35 (87.5)	35 (100)	0 (0)	0 (0)	0 (0)	35 (100)	34 (97.1)	33 (94.3)

### DNA damage of faeces cloned embryos

To clarify why cloned embryos failed to develop beyond the 2-cell stage, DNA damage of pronucleus was examined by a TUNEL assay. The male and female pronuclei of ICSI derived embryos and pronuclei derived from cumulus clones at the 1-cell stage was also examined as a control. As shown in Fig. 4B,C, the intensities of TUNEL assay in cloned embryos were significantly higher than cumulus cloned embryo and ICSI embryos (*P* < *0.01*). These results also suggest that failure of development of faeces cloned embryo was due to the DNA damage of original donor nuclei.

### Serial nuclear transfer aiming to the improvement of faeces cloned development

**Figure 5:**
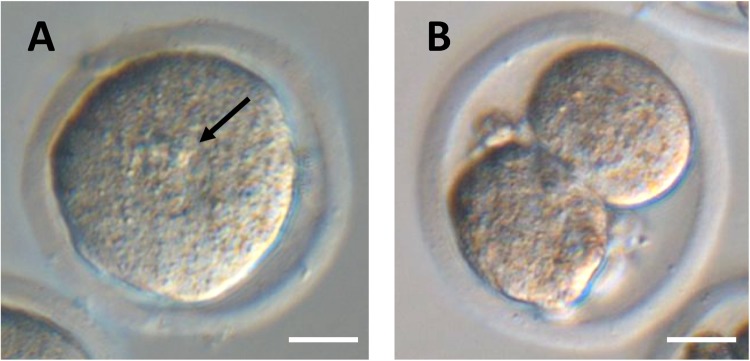
Pronuclear formation and development to the 2-cell stage of serial cloned embryos derived from faecal cell nuclei. Pronuclear formation of faeces-derived cloned zygote at 8 hr (**A**) and development to the 2-cell stage at 24 hr (**B**), when the second round of nuclear transfer was performed. Arrowhead indicates pronuclei. Bar = 20 μm.

To remove the toxic substances derived from faeces and enhance the DNA repair by oocytes cytoplasm, a serial nuclear transfer was performed. When the first round of nuclear transfer was performed, the toxic substances might be removed or diluted from donor nucleus inside oocytes cytoplasm. Then, cleaned donor nuclei were collected from those reconstructed oocytes and injected into other fresh oocytes as the second round of nuclear transfer. Theoretically, the toxic substances derived from faeces did not transfer into the second oocyte. In addition, the DNA damage of donor nuclei will be repaired two times by oocytes cytoplasm at the time of nuclear transfer. When the second round of nuclear transfer was performed, the rate of PN formation was 19.4%, which is comparable to the previous results (13–25%; Table 4) (Fig. 5A), and seven out of 18 (39%) cloned embryos developed to 2-cell stage (Fig. 5B). However, none of them developed beyond this stage, even cultured up to 4 days.

**Table 4:** Development of two-step faeces-dericed cell nuclear transferred embryos.

First round NT				Second round NT					
No. of NTs	Transferred nucleus positive (%)	Fragmented or nucleus negative (%)	Dead (%)	No. of NTs (%)	No. of PN formation (%)	No. of embryos development (%)			
						Frag. or 1-cell	2-cell	4–8 cell	Morulae/ blastocysts
204	96 (47.1)	100 (49.0)	8 (3.9)	93 (96.9)	18 (19.4)	85 (91.4)	7 (7.5)	0 (0)	0 (0)

## Discussion

In this study, we developed a cell collection method from faeces, and a relatively large number of faecal cells could be easily collected compare to urine-derived cells. Most likely, the origin of those cells was intestinal epithelial cells, and the surface area of intestine was much larger than bladder and urine tube; therefore, many intestinal cells that fell off were attached to the faeces. Thus, as the method for noninvasive cell collection, faeces were superior to urine.

Interestingly, some of those cell-like bodies (CLBs) derived from faeces did not have a nucleus. It is known that anucleated cells are not rare in epithelial cells (e.g., keratinocytes, vaginal epithelial cells in estrus and ureter epithelial cells)^12–14^. In spermiogenesis, there are cytoplasm-only structures in which the nucleus is discarded^15,16^. Although the origin of CLBs is uncertain, if the CLBs are from an intestinal epithelial cell, anucleated cells may also be observed in the faecal suspension^17^. Even nuclear-existing CLBs, more than half of cells did not express the GFP, which suggests that the membrane was damaged and GFP going out from cell cytoplasm. Because inside the intestines are a very harsh environment, with creeping movement and many digestive enzymes, it is very likely that the condition will cause damage to the cells until faeces are defecated. We note that some of faecal cells showed PI staining negative, which suggested that those faeces cells had intact cell membranes or were still alive. However, we could not find any sign of alive cells when cultured (unpublished observation).

When CLBs were injected into enucleated oocytes, only 4.2% of reconstructed oocytes formed pseudo-pronuclei after activation. When better donor cells were selected using several markers, the rate of PN formation increased up to 25%. However, unfortunately, none of the cloned embryos derived from faeces cell nuclei developed beyond the one-cell stage. When nuclear membrane, spindle and distribution of tubulin of cloned embryo/oocytes were examined, many abnormities were observed, which have never been seen in cumulus cell cloned embryo/oocytes. In addition, a developmental arrest happened only when the supernatant of the faecal suspension was injected into oocytes. It is known that faeces contain bile acid and endotoxins derived from intestinal bacteria^18,19^, and it is known that those substances cause a variety of damage such as DNA damage to the culture cells and embryos^20–23^. Most likely, the toxic substance of the faeces, which could not be removed from the cells by washing three times, was transferred into oocyte at the same time of nuclear transfer.

On the other hand, previously, Taberlet *et al*. reported that DNA isolated from faeces or noninvasive samples is often damaged^24^. We also confirmed that DNA of cloned embryos derived from faeces was extremely damaged compared to cumulus cell cloned embryos. Most likely, the toxic substance or creeping movement of intestines damaged not only cells but also its DNA. To overcome this obstacle, we planned to use the DNA repair capacity of oocytes. It is known that the oocyte cytoplasm has strong DNA repair potential, and healthy offspring were obtained from DNA damaged spermatozoa^25–28^. To enhance this process, we performed serial nuclear transfer procedure, which may enhance the DNA repair of damaged donor nuclei by repeating the nuclear transfer^29^. In addition, this procedure will also contribute to removing the donor-derived toxic substance because the donor nucleus was washed by the oocytes cytoplasm after the first round of nuclear transfer. Then, some recloned embryos could develop to the 2-cell stage, which has never been observed before. This procedure probably affected not only the repair of donor nuclei but also cleaned donor nuclei by repeating the nuclear transfer into oocytes cytoplasm. However, in this study, none of the cloned embryos developed beyond a 2-cell stage, which suggests that the DNA damage in most donor cell nuclei exceeded the DNA repair capacity of oocytes. Additional methods for efficient DNA damage repair is necessary to generate cloned mice from faecal cells.

In conclusion, we succeeded in establishing a collection method of donor cells from faeces and generating mouse-cloned embryos that developed to the 2-cell stage by using faecal-derived cells with serial cloning. If further effective cloning methods are established using faecal cells, e.g., completely removing toxic substances from faeces and repairing DNA damage, then full-term mouse development may be possible without any invasion of donor animal bodies, including endangered species.

## Materials and Methods

### Animals

Eight- to ten-week-old B6D2F1 (C57BL/6 × DBA/2 hybrid) female mice were purchased from Japan SLC (Shizuoka, Japan) and used as recipients for oocytes. The eGFP 129/Sv Tg (129-GFP) mouse strain was generated, maintained in our laboratory and used for the collection as donors for faeces-derived cells. The animals were housed under a controlled lighting condition (daily light 07:00–21:00 hr) and were maintained under specific-pathogen-free conditions. On the day of the experiments—or after having finished all experiments—mice were euthanized by CO_2_ inhalation or by cervical dislocation and used for experiments. All animal experiments were approved by the Institutional Committee of Laboratory Animal Experimentation at the University of Yamanashi and were performed in accordance with the Guide for the Care and Use of Laboratory Animals.

### Collection of cell-like bodies (CLBs)

Eight to ten pieces of mouse faeces were collected from the eGFP 129/Sv Tg males. The faeces were transferred to a 15 ml tube containing 1 ml PBS solution, and then, the tube was inverted a few times gently (Fig. 1A). Then, the faecal suspension of the tube was filtrated by 40 μm nylon filter (Cell Strainer, Becton, Dickinson and Company, NJ, USA) and washed two times by centrifugation (280 × g for 5 min). After centrifugation, the pellet was resuspended in PBS solution. The spherical cell-like form (Fig. 1B) was observed under the microscope (IX71; Olympus, Tokyo, Japan), and these forms were collected by micropipette attached with a micromanipulator. We refer to these forms as cells like bodies (CLBs).

### Isolation of CLBs from bacteria

Because there are numerous bacteria (e.g., *E. coli*) in faecal suspensions, it is necessary to remove bacteria from CLBs before use. Approximately 1–3 µl of the faeces-derived cell suspension was placed in a PVP medium droplet inside the micromanipulation chamber and mixed gently using sharp forceps. The CLBs were collected with a micropipette and moved into another PVP droplet. The body length of bacteria is approximately 0.2–5 µm, and it can be identified and excluded from CLBs when sucked into the micropipette under a Hoffman-modulated microscope. After confirmation that deposits of the same size as the bacteria were not present around the CLB, the CLB was injected into enucleated oocytes.

### Nuclear observation of CLBs

To detect whether CLBs were cells or not, the CLBs were costained with Hoechst 33342 (Sigma-Aldrich, MO, USA) and propidium iodide (PI; Sigma-Aldrich). These cells were observed under a fluorescence microscope (IX71), and the number of fluorescent cells was counted. Then, Hoechst-positive and PI-negative cells were judged as membrane intact cells, and Hoechst and PI double positive cells were judged as membrane damaged cells. In accordance with these observations, the presence or absence of GFP fluorescence was also confirmed.

### Collection of oocytes

B6D2F1 females were superovulated by the injection of 5 IU of pregnant mare serum gonadotropin (PMSG; Teikokuzoki, Tokyo, Japan) and 5 IU of human chorionic gonadotropin (hCG; Teikokuzoki) at a 48 hr interval. At 15 hr after hCG injection, the mice were euthanized, cumulus–oocyte complexes were collected from the oviducts, and the cumulus cells were removed by suspending them in HEPES-buffered CZB medium (H-CZB) containing 0.1% bovine testicular hyaluronidase (Sigma-Aldrich). After several minutes, the cumulus-free oocytes were washed and then moved to a droplet of CZB medium until use.

### Nuclear transfer (NT) of faeces-derived cell

The NT procedure was carried out as described earlier^30,31^, but the injection of donor nuclei was slightly modified^32^. Briefly, the donor nuclei were injected into enucleated oocytes using 8–10 μm of micropipette attached a Piezo-driven micropipette (PMM-150FU; Primetech, Ibaraki, Japan) in H-CZB containing 5 μg/ml cytochalasin B (Sigma-Aldrich). After injecting the donor nucleus into the oocyte, the membrane hole was pinched off from oocyte membrane. Those reconstructed oocytes were incubated in Ca^2+^-free CZB containing 3 mM SrCl_2_, 5 μM latrunculine A (Sigma-Aldrich) and 50 nM trichostatin A (TSA; Sigma-Aldrich) for 8 hr to activate oocytes and enhance the reprogramming. Then, those cloned embryos were cultured in CZB under 5% CO_2_ in air at 37 °C. For evaluation of cloned embryos development, the number of embryos that had reached the pronuclear and 2-cell stage after 8-hour post activation (hpa) and 24 hpa were counted and used in each assay. As a control, the cumulus cell nuclear transfer was also performed.

### Intracytoplasmic sperm injection (ICSI) and Parthenogenetic activation

As a control, fertilized embryos generated by ICSI and diploid parthenogenetic embryos were carried out as described earlier^33,34^. For ICSI, the spermatozoa were collected from 129-GFP mouse. The spermatozoa were suspended in a drop of 12% polyvinylpyrrolidone. A single sperm head was injected into metaphase II oocytes (B6D2F1) in a Hepes–CZB. The surviving oocytes after injection were cultured in CZB at 37 °C under 5% CO_2_ in air. For parthenogenetic embryos generation, oocytes were activated in Ca^2+^-free CZB containing 3 mM SrCl_2_ and 5 μg/ml cytochalasin B and then cultured for up to 6 hr in CZB containing 5 μg/ml cytochalasin B. After washing, the diploid parthenogenetic embryos were cultured in CZB at 37 °C in an atmosphere of 5% CO_2_ in air. These two type of embryos were observed in each stage or used in each assay.

### Observation of pseudo-pronuclear formation in reconstructed embryos by aceto-orcein staining and immunostaining

For aceto-orcein staining, the embryos were mounted on glass slides and fixed by 2.5% glutaraldehyde solution for 10 minutes. The fixed embryos were washed with water and 100% ethanol and then stained with 1 w/v % orcein solution (dissolved in 45% acetic acid solution). The slides were observed using a differential interference microscope (BX51; Olympus).

For immunostaining, the cloned embryos were stained with anti-Lamin B antibody and DAPI. The cloned embryos were fixed with 4% paraformaldehyde (PFA; Wako Pure Chemical, Osaka, Japan) at room temperature (RT) for 20 minutes. They were permeabilized with PBS solution containing 0.2% Triton X-100 (Sigma-Aldrich) at RT for 1 hr. They were then incubated with primary antibodies in PBS solution containing 30 mg/mL BSA at 4 °C overnight. After incubation, the embryos were reacted with secondary antibodies at RT for 1 hr. The primary antibodies were used as anti-lamin B (sc-6217; Santa Cruz Biotechnology, CA, USA) (1:500), The secondary antibodies were used as Alexa Fluor 568 donkey anti-goat IgG (H + L) (A11004; Thermo Fisher Scientific, PA, USA) (1:500). Specimens were mounted on glass slides in Vectashield mounting medium with DAPI (Vector Laboratories, CA, USA). The slides were then imaged using a laser scanning confocal microscope (FV1200; Olympus). As a control, cloned embryos derived from cumulus cell nuclei were used.

### Observation of tubulin distribution in cloned zygote

To observe the tubulin formation at 3 hr after donor nuclei injection, the reconstructed embryos were immunostained using beta-tubulin and DAPI. The staining method was same as described above. The primary antibodies were used as mouse anti-β-tubulin (BD556321; BD Biosciences, CA, USA) (1:10000). The secondary antibodies were used as Alexa Fluor 568 goat anti-mouse IgG (H + L) (A11004; Thermo Fisher Scientific) (1:500). As a control, reconstructed oocytes derived from cumulus cell nuclei and intact oocytes were used.

### Examination of the toxic of faeces substance

To detect the toxicity of faeces substance, approximately 10 pl of faeces suspension was injected into intact oocytes. As a control, faeces cell nuclei or PBS were also injected into intact oocytes. Those injected oocytes and noninjected oocytes were parthenogenetically activated as described above.

### Detection of apoptosis in cloned embryos

For detection of DNA damage, the pronuclear stage cloned embryos were examined by TUNEL assay. The cloned embryos at 8 hpa were fixed by 4% PFA and washed twice in PBS solution containing 30 mg/mL BSA; then, a TUNEL analysis was performed using *In Situ* Apoptosis Detection Kit (Merck Millipore, MA, USA) following the manufacturer’s recommendations. Specimens were mounted on glass slides in Vectashield mounting medium with DAPI. The slides were imaged using a laser scanning confocal microscope (FV1200) and then measured fluorescence intensity using an image processing software (ImageJ). As a control, reconstructed embryos derived from cumulus cell nuclei and ICSI embryos were used.

### Serial nuclear transfer

At 24 hr after nuclear transfer (first round NT), the nucleus was collected from those reconstructed oocytes and transferred into fresh enucleated oocytes (second round NT). The details of this method were described previously^29^. Those re-reconstructed oocytes were activated as described above.

### Statistical analysis

TUNEL intensity was analysed by one-way ANOVA analysis. A post hoc procedure using Tukey’s test was adopted for multiple comparisons between the groups (faeces-derived clone, cumulus clone and fertilized embryos). P values < 0.05 were considered significant.
